# Factors associated with exclusive breastfeeding practice among mothers in nine community health centres in Nanning city, China: a cross-sectional study

**DOI:** 10.1186/s13006-021-00416-x

**Published:** 2021-09-23

**Authors:** Jia Li, Chen Zhao, Yan Wang, Yin P. Wang, Chun Y. Chen, Yue Huang, Ya Q. Gao, Jin Fang, Hong Zhou

**Affiliations:** 1 School of Business, Nanjing University of Information Science & Technology, Nanjing, China; 2 China Development Research Foundation, Beijing, China; 3 Department of Maternal and Child Health, School of Public Health, Peking University, Beijing, China

**Keywords:** Exclusive breastfeeding, Early initiation of breastfeeding, Self-efficacy

## Abstract

**Background:**

The prevalence of exclusive breastfeeding (EBF) is suboptimal in China. There is limited evidence of effective interventions to increase EBF in China. Therefore, it is urgent to explore the potential factors that may be effective in promoting exclusive breastfeeding. Previous studies have mainly focused on socio-demographic factors and the Han ethnic group. This study explores more modifiable influencing factors of EBF in the Guangxi Zhuang Autonomous Region of China.

**Methods:**

The cross-sectional data used in this study were collected to provide baseline information on EBF prevalence for a breastfeeding promotion project. A total of 494 mothers of infants aged 0–5 months were recruited from nine community health centres in Nanning, China, in October 2019. Data were collected through face-to-face interviews using structured questionnaires. Infant feeding was measured by 24-h recall. The Chinese version of the Breastfeeding Self-Efficacy Scale–Short Form was used to examine the maternal breastfeeding self-efficacy. Univariate and multivariate logistic regressions were used to examine the factors associated with EBF practices.

**Results:**

In the present study, the prevalence of exclusive breastfeeding was 37.0%. Higher breastfeeding self-efficacy scores (adjusted odds ratio [AOR] 1.93; 95% confidence interval [CI] 1.25, 2.98), a college degree or above (AOR 2.15; 95% CI 1.24, 3.71), and early initiation of breastfeeding (AOR 2.06; 95% CI 1.29, 3.29) were positively associated with EBF practice. However, the preparation for infant formula before childbirth (AOR 0.30; 95% CI 0.17, 0.52) and premature birth (AOR 0.30; 95% CI 0.10, 0.87) were negatively associated with EBF practice.

**Conclusions:**

Exclusive breastfeeding practice was suboptimal and associated with various factors in the study area. The prevalence of EBF was positively associated with higher breastfeeding self-efficacy, education level of mothers, and early initiation of breastfeeding, whereas premature birth and preparation for infant formula before childbirth were barriers to exclusive breastfeeding. Future intervention projects should target mothers with premature babies, lower levels of education, and breastfeeding self-efficacy. Breastfeeding-friendly practices, such as the early initiation of breastfeeding and regulations on breastmilk substitutes, should also be encouraged.

## Background

Breastmilk is widely recognised as an optimal nutrition for infants, and breastfeeding provides short- and long-term health benefits for both infants and their mothers [1]. Given the demonstrated benefits of breastfeeding, the World Health Organization (WHO) and the United Nations Children’s Fund (UNICEF) recommend that mothers initiate breastfeeding within the first hour after birth, exclusively breastfeed their infants for the first six months, and continue breastfeeding until the child is two years of age or above [2, 3]. Nevertheless, breastfeeding practice remains suboptimal in China. Previous nationally representative surveys have shown a decreasing trend of exclusive breastfeeding (EBF) within the first six months in China over the past decade using internationally comparable indicators, with the rate dropping from 27.6% in 2008 to 20.7% in 2013 [4, 5].

Previous studies found that the suboptimal EBF practice in China was associated with a wide range of factors at individual, cultural, health facility, and socioeconomic levels, including maternal characteristics (such as age, education, employment status, ethnicity, and self-efficacy), child characteristics (such as age in months and premature birth), as well as practice, education, and support at health facilities (such as early initiation of breastfeeding and caesarean delivery) [6–14]. However, a majority of the previous studies in China have focused on specific regions and sometimes reached contradictory conclusions. For example, among 55 minority groups in China, some were found to share similar breastfeeding practices with the majority Han ethnic group [12], while other studies found that certain minority groups, such as the Uygur, Tibetan, and Zhuang, were different [15].

Recently, the China Development Research Foundation (CDRF) conducted a large-scale survey to provide a better understanding of the factors influencing breastfeeding among Chinese mothers between 2017 and 2018 [12]. Based on the factors identified in the large-scale survey, building a breastfeeding-friendly environment through successful intervention projects in health facilities is urgently needed [12]. Successful intervention projects to improve EBF in previous studies usually included education and support, targeting both the antenatal and postnatal periods, and combined multiple settings, such as hospitals and communities, in China and other countries [16–18]. Earlier intervention studies designed to promote EBF in China have mainly focused on breastfeeding education programs in a single setting, and tested the effectiveness of interventions among mothers from the Han ethnic group [19–23]. Little attention has been paid to comparatively modifiable factors, such as breastfeeding self-efficacy. Breastfeeding self-efficacy (BSE) is primarily defined as a mother’s confidence instead of her true ability to breastfeed her infant, and it has been extensively used in research on exclusive breastfeeding [24]. Furthermore, BSE may affect breastfeeding outcomes by providing mothers with the confidence to tackle common challenges, such as early latching difficulties and concerns of inadequate breastmilk supply [25]. Previous studies in Hong Kong and Guangzhou have disclosed a positive link between BSE and EBF among Chinese mothers [13, 14]. However, merely a few studies have tested this link in regions where minority groups are concentrated in China.

Additionally, the suboptimal EBF practice was closely related to national policy environments, such as maternity leave policy. Although the labour force participation rate of women in China ranks as one of the highest in the world [26], the length of maternity leave stipulated by the national government is only 98 days [27]. Each province mandates different maternity leave lengths ranging from 128 days to 1 year, while the majority allow less than the recommended duration of 6 months for EBF [28]. The influence of maternity leave on EBF is underexplored in China, especially in relation to the recent policy changes [29].

To fill these research gaps, the CDRF initiated a breastfeeding promotion project in Nanning, in the Guangxi Zhuang Autonomous Region in 2019, which had the largest ethnic minority population in China [30]. Combining both hospital and community settings, this project aimed to identify modifiable factors to improve EBF practice in a multi-ethnic context. The objective of the present study is to provide baseline information on breastfeeding practices and analyse the influencing factors of EBF practice using cross-sectional survey data, thereby providing implications for future health facility- and policy-level interventions designed to promote EBF in the study area.

## Methods

### Study design

The data were collected to provide baseline information for a breastfeeding promotion project in Nanning. Four hospitals and nine adjacent communities were selected as the pilot areas. This baseline survey was used to collect data on breastfeeding practices and their potentially associated factors for mothers of 0–5-month-old infants.

The four hospitals chosen in this project were all tertiary and Baby-Friendly Hospital Initiative (BFHI)-certified hospitals in Nanning. They were an ideal setting for recruiting women from different socio-demographic backgrounds. Nine communities were recommended by the hospitals based on the principle of geographical proximity and the overlapping of the health service radius between the hospitals and communities.

### Study variables

We used indicators recommended by the WHO to assess infant feeding practices based mainly on food and drink consumed in the past 24 h. The EBF rate, that is, the proportion of 0–5-month-old infants who were fed exclusively with breastmilk in the past 24 h (no foods or liquids except for drops and syrups), was the key indicator in the present study [31]. In addition to the EBF rate, based on the definitions recommended by the WHO, we also calculated the rates of predominant breastfeeding, complementary feeding, and no breastfeeding. The predominant breastfeeding rate was calculated as the proportion of 0–5-month-old infants who were fed with breastmilk and certain liquids, such as water, water-based drinks, and fruit juice, in the past 24 h. The complementary feeding rate was calculated as the proportion of 0–5-month-old infants who were fed with breastmilk and any food or liquid, including formula and non-human milk, in the past 24 h [32]. No breastfeeding rate was calculated as the proportion of 0–5-month-old infants who were not breastfed.

The selection of potential EBF risk factors was based on a review of the previous literature [11], including maternal characteristics (age, ethnicity, education, and formal employment status), child characteristics (age in months, gender, having older siblings, premature birth, and low birthweight), health facility practices (caesarean delivery and early initiation of breastfeeding), exposure to breastmilk substitutes (preparation for infant formula before childbirth), breastfeeding-related experiences (any breastfeeding difficulties in the postnatal period and breastfeeding self-efficacy), and the length of maternity leave. Premature birth indicated infants born before 37 weeks of age. Low birthweight was defined as a birthweight less than 2500 g. Early initiation of breastfeeding referred to the practice of infants being put to their mother’s breast within one hour of birth. Preparation for infant formula before childbirth meant that mothers bought infant formula regardless of their intentions to use it or not before childbirth. Furthermore, BSE was assessed with the Breastfeeding Self-Efficacy Scale–Short Form (BSES-SF), which consists of 14 questions on a 5-point Likert scale, where 1 indicates *not at all confident* and 5 indicates *always confident* [24]. All items are displayed positively, and all scores are summed to produce a total score from 14 to 70, with higher scores indicating higher BSE levels. The Chinese translation of the BSES-SF was validated in the previous literature, where the Cronbach’s alpha coefficient for internal consistency was larger than 0.9, and its unidimensional structure was justified via confirmatory factor analysis [13, 22, 33]. Following the previous literature, we considered mothers as having high BSE if their total scores on the BSES-SF were 55 or higher [34].

### Sample size

The present study was designed to collect baseline information on the EBF practice and its associated factors. The assumed EBF baseline prevalence was 26.7% (*p*_*0*_) based on data previously collected in Nanning by the CDRF and the National Institute for Nutrition and Health of the Chinese Centre for Disease Control and Prevention from September 2017 to January 2018 [12]. The maximum affordable sample size was 500 mothers due to the budget and time constraints of the present study. With a sample size of 500 mothers, a 95% confidence interval was expected to have a relative precision of 15% [35].

### Participants

The survey used a stratified random sampling methodology. Mothers of infants under six months were stratified by community, and each stratum consisted of one community health centre.

### Sampling

The sample size for each stratum was proportional to its size, that is, the total number of eligible mothers. At each community health centre, we randomly selected mothers of 0–5-month-old infants in the immunisation clinic waiting areas before vaccination during the survey period until the desired sample size was obtained. Owing to the large number of infants, the mothers usually had to wait for more than 30 min before their turns, and the number of available seats for mothers to sit down during the waiting time was insufficient. In collaboration with the immunisation clinics, we opened a green channel to enable mothers who participated in the survey to get vaccinated immediately after data collection. We also set up a relatively independent space for the interviewers to ensure the quality of data collection in each community health centre. In total, 494 mothers of infants under six months were included in the cross-sectional survey.

### Inclusion and exclusion criteria

The inclusion criteria were mothers of 0–5-month-old infants who delivered their babies in one of the four hospitals and also lived in one of the nine communities, signed the informed consent form, had no psychiatric disorders, and were able to answer the questions clearly. Psychiatric disorders indicated mental illness, as diagnosed by mental health professionals. Answering the questions clearly meant that mothers could understand and respond to the questions fluently in Mandarin Chinese. The exclusion criteria were mothers who were not the primary caregivers of their babies or who had psychiatric disorders.

### Data collection

The survey was conducted by an independent research team from the School of Public Health at Peking University. Data were collected through face-to-face interviewer-administered questionnaires. The questionnaire used in the present study was structured and developed by researchers from Peking University and the CDRF breastfeeding team. The questionnaire covered the socio-demographic information of the mothers, the length of maternity leave, breastfeeding practices, health facility practices, and breastfeeding self-efficacy.

Data were collected by 27 enumerators in October 2019. The team of enumerators was recruited from nine community health centres and Guangxi Medical University and trained in Nanning. The enumerator selection criteria included education and training background in public health as well as fluency in Mandarin. A half-day enumerator training session was organised by the same team from Peking University. The training sessions consisted of an explanation of the survey, instructions regarding interviewing techniques and filing procedures, a detailed review and explanation of the questionnaire items, a discussion on the questionnaire items, one-on-one mock interviews, and interview practice. Only those who could fill out the questionnaire with a 90% accuracy or above were considered as qualified enumerators.

Eight researchers and staff from Peking University and the CDRF served as field supervisors and coordinators to oversee the standardised data collection procedure and perform quality control. The study design was approved by the Medical Research and Clinical Trial Ethics Committee at the Maternal and Child Health Hospital of the Guangxi Zhuang Autonomous Region (approval number: 2019–02). Written consent was obtained from all mothers.

### Data analysis

This study first used a descriptive analysis to report the demographic information of the survey participants by EBF status and assessed their associations using the chi-square test. Demographic information included maternal and child characteristics. Maternal characteristics included age (< 35 and > = 35), ethnicity (Han and others), education level (high school or below and college or above), and whether the mother was formally employed (yes or no). Infant characteristics included age in months, gender (male or female), whether the infants had older siblings (yes or no), premature birth (yes or no), and low birthweight (yes or no). Health facility practices included caesarean delivery (yes or no) and early initiation of breastfeeding (yes or no). Exposure to breastmilk substitutes included the preparation for infant formula before childbirth. Breastfeeding-related experiences consisted of any breastfeeding difficulties in the postnatal period (yes or no) and BSE score (< = 55 or > = 56). The length of maternity leave was classified into two categories: 0–98 days and > 98 days. Thereafter, we calculated the percentage distribution of infant feeding practices by age in months using the indicators mentioned earlier. Lastly, univariate and multivariate logistic regressions were conducted to determine the unadjusted and adjusted odds ratios of the EBF risk factors.

From the 494 mothers interviewed, we excluded one due to incomplete information on breastfeeding practices. We also excluded 17 observations with missing values for the covariate variables (e.g., age of the mother, early initiation of breastfeeding, low birthweight, gender of the child, and whether the mother prepared for infant formula before childbirth). Thus, the final sample size for statistical analysis was 476, which was more than 96% of the original sample size. A sensitivity analysis using the full sample showed similar results. All data analyses were performed using STATA version 14.1 (Stata Corporation, USA). We set 0.05 as the statistical significance.

## Results

### Sample description

**Fig. 1:**
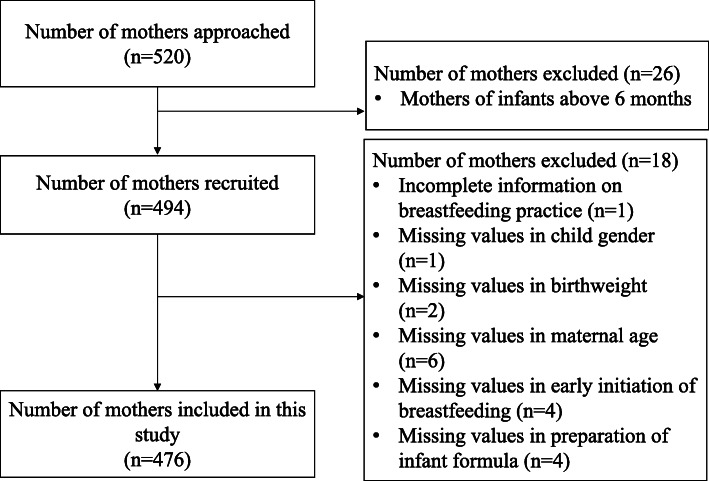
Flow diagram for selection of analytic sample of mothers

In the study area, the estimated number of eligible mothers was 4831, based on the data submitted by the nine health community centres prior to the survey. We approached 520 mothers, among which 494 were mothers of infants under six months old (Fig. 1). Owing to the missing values in the covariate factors, we only included 476 mothers in our study, among which 20.2% were above the age of 35, 56.5% belonged to the Han ethnicity, 65.1% had a college education or above, and 52.9% were formally employed (Table 1). A statistically significant difference was observed in the educational level among mothers who practiced EBF and those who did not. Similar distributions of age and ethnicity were observed between the two groups of mothers.

**Table 1:** Influence of mother, infant and other breastfeeding related characteristics on EBF practice (*n* = 476)

Risk factors	Total		EBF		Non-EBF		***X***^***2***^	***df***^**a**^	***P***-value^**b**^
	N	%	n	%	n	%			
**Maternal characteristics**									
Age:									
< 35	380	79.83	147	83.52	233	77.67	2.36	1	0.12
> = 35	96	20.17	29	16.48	67	22.33			
Ethnicity:									
Others	207	43.49	80	45.45	127	42.33	0.44	1	0.51
Han	269	56.51	96	54.55	173	57.67			
Education level:									
High school or below	166	34.87	43	24.43	123	41.00	13.41	1	0.00
College or above	310	65.13	133	75.57	177	59.00			
Formal employment:									
No	224	47.06	73	41.48	151	50.33	3.49	1	0.06
Yes	252	52.94	103	58.52	149	49.67			
**Child characteristics**									
Age in months:							23.72	1	0.00
0	62	13.03	35	19.89	27	9.00			
1	106	22.27	42	23.86	64	21.33			
2	82	17.23	39	22.16	43	14.33			
3	107	22.48	33	18.75	74	24.67			
4	64	13.45	19	10.80	45	15.00			
5	55	11.55	8	4.55	47	15.67			
Gender:									
female	213	44.75	82	46.59	131	43.67	0.38	1	0.54
male	263	55.25	94	53.41	169	56.33			
Have older siblings:									
No	230	48.32	87	49.43	143	47.67	0.14	1	0.71
Yes	246	51.68	89	50.57	157	52.33			
Premature birth:									
No	432	90.76	171	97.16	261	87.00	2.90	1	0.00
Yes	44	9.24	5	2.84	39	13.00			
Low birthweight:									
No	437	91.81	168	95.45	269	89.67	13.65	1	0.03
Yes	39	8.19	8	4.55	31	10.33			
**Health facility practice**									
Caesarean delivery:									
No	332	69.75	131	74.43	201	67.00	4.94	1	0.09
Yes	144	30.25	45	25.57	99	33.00			
Early initiation of breastfeeding:									
No	254	53.36	70	39.77	184	61.33	20.72	1	0.00
Yes	222	46.64	106	60.23	116	38.67			
**Exposure to breastmilk substitutes**									
Prepared for infant formula before childbirth:									
No	85	17.86	48	27.27	37	12.33	16.88	1	0.00
Yes	391	82.14	128	72.73	263	87.67			
**Breastfeeding experience**									
Any breastfeeding difficulties in postnatal period:									
No	228	47.90	99	56.25	129	43.00	7.80	1	0.01
Yes	248	52.10	77	43.75	171	57.00			
Breastfeeding self-efficacy score:									
< =55	301	63.24	98	55.68	203	67.67	6.85	1	0.01
> =56	175	36.76	78	44.32	97	32.33			
**Length of maternity leave:**									
0–98 days	261	54.83	85	48.30	176	58.67	4.82	1	0.03
> 98 days	215	45.17	91	51.70	124	41.33			

^a^ Degree of freedom. ^b^*P*-value was based on chi-square test

Regarding infant characteristics, 55.3% of the infants were boys, and more than half had older siblings. The prevalence of EBF was similar for boys (35.7%) and girls (38.5%), and for infants who had or did not have older siblings. In total, 9.2% and 8.2% of them were premature births or had low birthweights, respectively. The proportions of premature births (*P* < 0.001) and low birthweights (*P* = 0.026) were significantly lower among mothers who practiced exclusive breastfeeding.

Over 30% of the infants were born via caesarean delivery. Early initiation of breastfeeding was practiced by 46.6% of mothers. Mothers who initiated breastfeeding earlier (*P* < 0.001) were more likely to exclusively breastfeed their infants.

More than half of the mothers had breastfeeding difficulties after discharge, and approximately 82% prepared for infant formula before childbirth. For mothers’ BSE scores, only 36.8% were above the threshold of 55 points to be considered as having high breastfeeding self-efficacy. Mothers who had a high self-efficacy score (*P* = 0.009) were more likely to exclusively breastfeed their infants, whereas mothers who reported breastfeeding difficulties postpartum (*P* = 0.005) or who prepared for infant formula before childbirth (*P* < 0.001) had a lower likelihood of practicing exclusive breastfeeding.

Approximately 45.2% of mothers had more than 98 days of maternity leave. Among mothers with a longer length of maternity leave, the likelihood of practicing EBF was significantly higher than that of mothers with less than 98 days of maternity leave (*P* = 0.028).

### Percentage distribution of breastfeeding practice

**Fig. 2:**
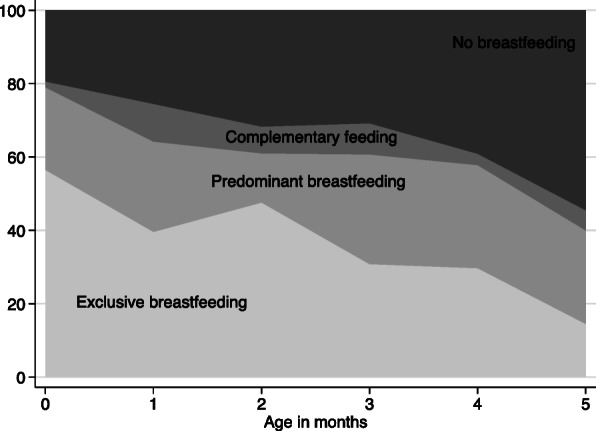
Percent distribution of breastfeeding practices of infants within 6 months (*n* = 476). Note: Calculation of breastfeeding practice was based on the 24-h recall standard itemized checklist of the WHO

Figure 2 shows the percentage distribution of breastfeeding practices in the mothers by age in months of infants. The prevalence of EBF under six months was 37.0% in our sample, which dropped from 41.7% at 0–3 months to 29.7% at four months and then to 14.5% at five months (Fig. 2). Predominant breastfeeding (mainly including feeding plain water) and no breastfeeding were the main barriers to exclusive breastfeeding. The prevalence of predominant breastfeeding did not change significantly with age in months, while the proportion of no breastfeeding significantly increased from 27.7% at 0–3 months to 47.6% at 4–5 months (Fig. 2).

### Factors influencing exclusive breastfeeding

Table 2 displays the factors that are significantly associated with EBF practice with both unadjusted and adjusted odds ratios as well as 95% CIs. We adjusted for all potential risk factors shown in Table 1. After adjusting for other potential confounders, we found that mothers with a college degree or above were more likely to breastfeed their children exclusively (AOR 2.15; 95% CI 1.24, 3.71). The odds of EBF were lower for mothers with premature babies (AOR 0.30; 95% CI 0.10, 0.87) than those with full-term babies and mothers who prepared for infant formula before childbirth (AOR 0.30; 95% CI 0.17, 0.52). Mothers who initiated breastfeeding early had a significantly higher chance of practicing exclusive breastfeeding (AOR 2.06; 95% CI 1.29, 3.29). Additionally, the association of BSE scores with EBF showed that mothers with higher scores (≥ 56) were more likely to practice EBF than those with lower scores (AOR 1.93; 95% CI1.25, 2.98).

**Table 2:** Risk factors for mothers of 0–5-month-old infants who practiced EBF – unadjusted and adjusted Odds Ratio (*n* = 476)

	EBF		Unadjusted		Adjusted	
Variable	n	%^a^	OR^b^	95%CI^c^	OR^d^	95%CI
**Maternal characteristics**						
Age:						
< 35	380	38.7	1		1	
> = 35	96	30.2	0.69	(0.42–1.11)	0.61	(0.34–1.10)
Ethnicity:						
Others	207	38.6	1		1	
Han	269	35.7	0.88	(0.61–1.28)	0.77	(0.51–1.18)
Education level:						
High school or below	166	25.9	1		1	
College or above	310	42.9	2.15***	(1.42, 3.25)	2.15**	(1.24–3.71)
Formal employment:						
No	224	32.6	1		1	
Yes	252	40.9	1.43	(0.98, 2.08)	1.11	(0.51–2.41)
**Child characteristics**						
Age in months			0.73***	(0.64, 0.83)	0.74***	(0.64–0.85)
Gender:						
female	213	38.5	1		1	
male	263	35.7	0.89	(0.61–1.29)	0.88	(0.58–1.34)
Have older siblings:						
No	230	37.8	1		1	
Yes	246	36.2	0.93	(0.64–1.35)	0.85	(0.55–1.34)
Premature birth:						
No	432	39.6	1		1	
Yes	44	11.4	0.20***	(0.08, 0.51)	0.30*	(0.10–0.87)
Low birthweight:						
No	437	38.4	1		1	
Yes	39	20.5	0.41*	(0.19, 0.92)	0.72	(0.27–1.95)
**Health facility practice**						
Caesarean delivery:						
No	332	39.5	1		1	
Yes	144	31.3	0.70	(0.46–1.06)	1.49	(0.87–2.53)
Early initiation of breastfeeding:						
No	254	27.6	1		1	
Yes	222	47.7	2.40***	(1.64, 3.52)	2.06**	(1.29–3.29)
**Exposure to breastmilk substitutes**						
Prepared for infant formula before childbirth:						
No	85	56.5	1		1	
Yes	391	32.7	0.38***	(0.23, 0.61)	0.30***	(0.17–0.52)
**Breastfeeding experience**						
Any breastfeeding difficulties postpartum:						
No	228	43.4	1		1	
Yes	248	31.0	0.59**	(0.40, 0.85)	0.77	(0.51–1.18)
Breastfeeding self-efficacy score:						
< =55	301	32.6	1		1	
> =56	175	44.6	1.67**	(1.14, 2.44)	1.93**	(1.25–2.98)
**Length of maternity leave**:						
0–98 days	261	32.6	1		1	
> 98 days	215	42.3	1.52*	(1.04, 2.21)	1.00	(0.47–2.11)

^a^ Percentage of mothers who practiced EBF. ^b^ Odds Ratio (OR). ^c^ Confidence interval (CI). *** *p* < 0.001, ** *p* < 0.01, * *p* < 0.05. ^d^ Adjusted for all the variables in Table 1

Furthermore, the distribution of EBF varies at different ages in months, as shown in Fig. 2. Thus, we classified the sample into two subgroups: mothers of 0–2-month-old and 3–5-month-old infants to examine their predictors of exclusive breastfeeding. Table 3 displays the unadjusted and adjusted odds ratios and 95% CIs for each subgroup. Among the mothers of the 0–2-month-old infants, we found that the odds of EBF were higher among mothers who initiated breastfeeding early (AOR 1.95; 95% CI 1.08, 3.54), had an education level of college or above (AOR 2.04; 95% CI 1.00, 4.15), and had a higher self-efficacy score (AOR 1.86; 95% CI 1.05, 3.30).

**Table 3:** Risk factors of 0–5-month-old infants who practiced EBF by subgroups

	(1)	(2)	(3)	(4)	(5)	(6)	(7)	(8)
	0–2 months				3–5 months			
	Unadjusted		Adjusted		Unadjusted		Adjusted	
**Variable**	**OR**^**a**^	**95%CI**^**b**^	**OR**^**c**^	**95%CI**	**OR**^**a**^	**95%CI**^**b**^	**OR**^**c**^	**95%CI**
**Maternal characteristics**								
Age:								
< 35	1		1		1		1	
> = 35	0.77	(0.42–1.39)	0.69	(0.34–1.42)	0.37*	(0.14–0.99)	0.49	(0.16–1.47)
Ethnicity:								
Others	1		1		1		1	
Han	0.80	(0.49–1.33)	0.76	(0.44–1.32)	0.96	(0.53–1.74)	0.90	(0.46–1.77)
Education level:								
High school or below	1		1		1		1	
College or above	2.00*	(1.18–3.41)	2.04*	(1.00–4.15)	2.87**	(1.39–5.92)	2.24	(0.89–5.64)
Formal employment:								
No	1		1		1		1	
Yes	1.38	(0.84–2.29)	1.45	(0.54–3.86)	1.41	(0.78–2.55)	0.85	(0.21–3.40)
**Child characteristics**								
Gender:								
female	1		1		1		1	
male	1.20	(0.73–1.98)	1.15	(0.66–1.98)	0.62	(0.34–1.12)	0.65	(0.32–1.28)
Have older siblings:								
No								
Yes	1.04	(0.63–1.72)	0.97	(0.54–1.72)	0.61	(0.33–1.11)	0.73	(0.35–1.51)
Premature birth:								
No	1		1		1		1	
Yes	0.44	(0.14–1.45)	0.75	(0.20–2.82)	0.08*	(0.01–0.60)	0.06*	(0.01–0.66)
Low birthweight:								
No	1		1		1		1	
Yes	0.37	(0.10–1.40)	0.50	(0.11–2.18)	0.60	(0.21–1.65)	1.29	(0.31–5.48)
**Health facility practice**								
Caesarean delivery:								
No	1		1		1		1	
Yes	0.72	(0.41–1.27)	1.31	(0.65–2.62)	0.74	(0.39–1.40)	1.94	(0.82–4.62)
Early initiation of breastfeeding:								
No	1		1		1		1	
Yes	2.07**	(1.25–3.43)	1.95*	(1.08–3.54)	2.61**	(1.42–4.77)	2.26*	(1.02–5.00)
**Exposure to breastmilk substitutes**								
Prepared for infant formula before childbirth:								
No	1		1		1		1	
Yes	0.37**	(0.19–0.72)	0.34**	(0.16–0.72)	0.37**	(0.18–0.76)	0.24**	(0.09–0.61)
**Breastfeeding experience**								
Any breastfeeding difficulties postpartum:								
No	1		1		1		1	
Yes	0.63	(0.38–1.04)	0.64	(0.37–1.12)	0.59	(0.33–1.07)	0.77	(0.39–1.52)
Breastfeeding self-efficacy score:								
< =55	1		1		1		1	
> =56	1.50	(0.89–2.50)	1.86*	(1.05–3.30)	1.99*	(1.09–3.64)	2.00	(0.99–4.04)
**Length of maternity leave:**								
0–98 days	1		1		1		1	
> 98 days	1.45	(0.88–2.39)	0.83	(0.32–2.17)	1.58	(0.87–2.86)	1.20	(0.31–4.59)

^a^ Odds Ratio (OR). ^b^ Confidence interval (CI). *** *p* < 0.001, ** *p* < 0.01, * *p* < 0.05. ^c^ Adjusted for all the variables in Table 1

However, the prevalence of EBF was lower among mothers who prepared for infant formula before childbirth (AOR 0.34; 95% CI0.16, 0.72). Among the mothers of the 3–5-month-old infants, in addition to similar predictors including early initiation of breastfeeding (AOR 2.26; 95% CI 1.02, 5.00) and preparation for infant formula before childbirth (AOR 0.24; 95% CI0.09, 0.61), premature birth was also negatively associated with exclusive breastfeeding (AOR 0.06; 95% CI 0.01, 0.66).

## Discussion

In the present study, we found that breastfeeding practice was suboptimal in nine community health centres in Nanning, China, and there was no significant difference in EBF practice between Han and other minority groups. The prevalence of EBF under six months in our study (37.0%) was higher than the national prevalence reported in 2013 (20.7%; crude) [4] but lower than the national target of 50% [36]. The comparatively higher prevalence of EBF in our sample was possibly due to the purposive selection of hospitals. All the hospitals selected for this survey are BFHI-certified hospitals, which are among the best hospitals in Nanning [37]. The BFHI was launched by the Chinese Ministry of Health in collaboration with the WHO and UNICEF in 1991 to protect, promote, and support breastfeeding in maternity facilities. A series of baby-friendly policies and practices, such as the ‘Ten Steps to Successful Breastfeeding’ and the relevant regulations of the *International Code of Marketing of Breastmilk Substitutes* [38], have been adopted in BFHI-certified hospitals. Strengthening and sustaining baby-friendly policies and practices is essential to increase EBF, especially in hospitals in China.

The percentage distribution of breastfeeding practices in Fig. 2 revealed that the prevalence of EBF decreased sharply from the fourth month postpartum in the present study. This was likely due to the maternity leave policy in the study area. The local policy extended the length of maternity leave to 148 days in 2016 [39], and 45.2% of mothers in our sample were entitled to more than 98 days of maternity leave. Mothers with more than 98 days of maternity leave were 10% more likely to practice EBF than those with less. Thus, the extended maternity leave in the study area might partially explain the better EBF performance [40]. In the multiple logistic regression, after controlling for the education level of mothers, the length of maternity leave was not significantly associated with EBF practice (AOR 1.00; 95% CI 0.47, 2.11). More studies are needed to clarify the influence of longer maternity leave on EBF practice in China.

In the present study, we also found that the prevalence of EBF was significantly associated with maternal and child characteristics, health facility practices, the BSE of mothers, and the preparation for infant formula before childbirth.

First, regarding maternal characteristics, education level was identified as an influencing factor for EBF practice in the present study. Mothers with a college degree or above were associated with a higher prevalence of EBF, which was consistent with previous studies in developed countries [41, 42], however, this finding contradicted the results of an earlier systematic review in China [43]. The negative or non-significant associations between maternal education and EBF observed in previous studies may be due to the shortness of the maternity leave among mothers with higher education levels [43]. However, the considerably longer maternity leave in our study area enabled mothers to breastfeed for a longer time. Additionally, mothers with higher education levels may practice EBF because they are more knowledgeable about the definition and benefit of EBF, as suggested by a previous study in Indonesia [44]. Breastfeeding education sessions may need to target mothers with lower education levels to improve their knowledge of breastfeeding.

Second, regarding child characteristics, we reported that premature birth was inversely related to EBF practice. This is possibly due to the dearth of support for initiating and establishing lactation to mothers with premature infants during their hospital stay, which complicates their continuation of breastfeeding after discharge. A previous study found that knowledge about breastfeeding premature infants among neonatal intensive care unit (NICU) healthcare providers was limited in mainland China [45]. To support mothers of preterm infants in successfully initiating and continuing breastfeeding, NICU healthcare providers may need more targeted training to improve their knowledge of breastfeeding premature infants.

Third, regarding health facility practices, our study confirmed that the early initiation of breastfeeding had a positive effect on EBF practice. The prevalence of early initiation of breastfeeding in our sample was 46.6%, and over half of the mothers did not initiate breastfeeding within an hour of birth [46]. The lower prevalence of early initiation of breastfeeding may be due to the high prevalence of caesarean deliveries in the study area. Previous studies have suggested that caesarean deliveries are significantly associated with unsuccessful and delayed breastfeeding [47]. In our sample, the prevalence of early initiation of breastfeeding was 14.6% among mothers who delivered their babies by caesarean sections, which was much lower than that of 60.5% among mothers who delivered their babies through natural childbirth. Another reason for the low rate of early initiation may be the lack of knowledge about early initiation of breastfeeding [6]. The government and hospitals should take effective measures to reduce the prevalence of caesarean sections. Hospitals also need to strengthen education on the early initiation of breastfeeding, improve the implementation of the ‘Ten Steps to Successful Breastfeeding’ to help mothers initiate breastfeeding immediately after childbirth, and provide special support for mothers who deliver via caesarean section [38].

Fourth, our results also revealed that mothers with high BSE scores were more likely to breastfeed their children exclusively, which conformed with previous studies [22, 48]. The reason why higher BSE levels are related to a higher EBF prevalence is that mothers with higher levels of BSE are inclined to be more positive and willing to make an effort to solve breastfeeding problems [49]. The average BSE score assessed by the BSES-SF in our study was 52.3, which was higher than that in Chinese studies in Wuhan (48.1) [22], Hong Kong (41.1) [33], and Guangdong (47.3) [13]. Unlike socio-demographic factors, BSE has been identified as a possible modifiable factor [50, 51]. Previous studies in China suggested that BSE was influenced by social support (such as from husbands and healthcare providers), early initiation of breastfeeding, and attending antenatal breastfeeding classes [52, 53]. Social support provided various items of information that influenced mothers to choose, perform, and maintain breastfeeding behaviour through vicarious experiences and verbal persuasion [49]. However, verbal persuasion with misperceptions may be negatively associated with optimal breastfeeding practices. For instance, over 80% of the mothers prepared for infant formula before childbirth, and approximately 41% were advised by their family members, which was in agreement with a previous study in another country [54]. The regression results in this study suggested that mothers who prepared for infant formula before childbirth were less likely to practice exclusive breastfeeding. On the one hand, being told to prepare for infant formula by family members may undermine the BSE of mothers. On the other hand, mothers who prepared for infant formula by themselves may have lacked confidence in breastfeeding. In China, a recent study conducted in Wuhan showed that an individualised intervention comprising assessment, self-efficacy-enhancing strategies, and evaluation could reduce perceptions of insufficient milk supply and thereby improve BSE and EBF in a hospital setting during the postpartum period [22]. However, community-based interventions in the postpartum period are scarce in China. In our sample, approximately 22% of mothers with higher BSE had received breastfeeding consultation at a community health centre, which was higher than the 16% of mothers with lower breastfeeding self-efficacy. Considering that BSE is only significantly associated with EBF for mothers of 0–3-month-old infants, more intervention studies are needed to assess the most effective ways to improve BSE in both hospital and community settings in China, especially in the early stages after childbirth. Future intervention studies may also need to correct common breastfeeding misperceptions among family members to reduce their negative influence on the BSE of mothers.

### Limitations

There are several limitations to the present study. First, being a cross-sectional study; we collected data on mothers’ feeding practices in the past 24 h and experiences before and immediately after childbirth. The data collected in this study may suffer from recall bias, especially for mothers with older infants. Second, the cross-sectional nature of the data prevented us from determining the causal relationships of various factors with EBF. Third, the purposive selection of hospitals and community health centres in our survey resulted in a population with a comparatively high education level. Thus, the results cannot be generalised to the whole of Nanning. Fourth, owing to budget and time constraints, we applied convenience sampling at the immunisation clinics of community health centres. Given that the coverage of the immunisation registry and immunisation program is 99% in China, the survey participants may reflect the majority of the entire population [55]. However, because we limited the respondents to mothers, children who were not with their mothers at the time of the survey were excluded. Thus, our results may overestimate the breastfeeding situation in the nine community health centres.

## Conclusions

To sum up, we found that breastfeeding practice was suboptimal in nine community health centres in Nanning, China, and was associated with various factors. The associations found in the present study have important implications for future interventions designed to provide breastfeeding education and support in both hospital and community settings in the Guangxi Zhuang Autonomous Region of China. Regarding health facility practices, given the benefits of immediate and continuous mother–infant skin-to-skin contact and first latch, it is essential to improve the prevalence of early initiation of breastfeeding to promote EBF in China, especially among premature births and infants delivered by caesarean section. Second, breastfeeding education sessions need to correct common misperceptions of breastfeeding involving both mothers and their family members, especially for those with lower education levels. Third, future breastfeeding interventions may need to focus more on modifiable factors, such as self-efficacy, to improve the breastfeeding outcomes of mothers in the postnatal period. Fourth, breastfeeding-friendly policies, such as optimal lengths of maternity leaves, may need to be actively adopted in China.

## Data Availability

The dataset is available from the corresponding author on reasonable request.

## Abbreviations

BFHI: Baby-Friendly Hospital Initiative
BSE: Breastfeeding Self-efficacy
BSES-SF: Breastfeeding Self-Efficacy Scale (Short form)
CDRF: China Development Research Foundation
EBF: Exclusive Breastfeeding
NICU: Neonatal Intensive Care Unit
UNICEF: United Nations Children’s Fund
WHO: World Health Organization
